# COVID-19 in Pediatric Patients: A Focus on CHD Patients

**DOI:** 10.3389/fcvm.2020.612460

**Published:** 2020-11-27

**Authors:** Rana O. Zareef, Nour K. Younis, Fadi Bitar, Ali H. Eid, Mariam Arabi

**Affiliations:** ^1^Faculty of Medicine, American University of Beirut Medical Center, Beirut, Lebanon; ^2^Division of Pediatric Cardiology, Pediatric Department, American University of Beirut Medical Center, Beirut, Lebanon; ^3^Department of Basic Medical Sciences, College of Medicine, QU Health, Qatar University, Doha, Qatar; ^4^Biomedical and Pharmaceutical Research Unit, QU Health, Qatar University, Doha, Qatar; ^5^Department of Pharmacology and Toxicology, American University of Beirut, Beirut, Lebanon

**Keywords:** children, congenital heart disease, CHD, COVID-19, coronavirus, pediatric cardiology

## Abstract

Coronavirus disease 2019 (COVID-19) is a global pandemic caused by SARS-CoV-2 virus. As of the 30th of September 2020, around 34,000,000 cases have been reported globally. Pediatrics with underlying congenital heart disease represent a small yet a critical proportion of these patients. In general, the majority of infected children experience mild to moderate disease with significant interindividual variability in laboratory and radiographic findings. Nevertheless, in healthy children with COVID-19, cardiac involvement has been documented and is attributed to various causes. Myocarditis, arrhythmias, cardiogenic shock, and serious multisystem inflammatory syndrome in children are all encountered. Since COVID-19 is a recent novel disease and based on previous experience with respiratory infections, children with underlying congenital heart disease should be given special attention. To date, little data is available about COVID-19 presentation, complications, and appropriate treatment in this population. However, variable and inconsistent disease presentation and severity have been observed. This paper discusses COVID-19 course of illness in pediatric population with a special emphasis on the cardiac manifestations of the disease in healthy population and also on the disease course in congenital heart disease patients in particular.

## Introduction

Coronaviruses (CoVs) are enveloped single stranded RNA viruses that belong to the Coronaviridae family (1). They are implicated in a wide spectrum of diseases ranging from mild illness such as common cold to more serious life-threatening syndromes such as the Middle East Respiratory Syndrome (MERS) and the Severe Acute Respiratory Syndrome (SARS) (2). Toward the end of 2019, a novel coronavirus, called SARS-CoV-2, led to the unprecedented pandemic of Coronavirus Disease 2019 (COVID-19) (3). At the time of writing this manuscript, the total COVID-19 cases reported are around 34 million, with over 1 million deaths reported.

SARS-CoV-2 infection is not only associated with respiratory symptoms but also with multi-organ manifestations that include cardiac, gastrointestinal, hematologic, renal, and neurologic ones (4–7). Despite the rapid global spread of the pandemic, the disease characteristics in pediatrics with congenital heart disease (CHD) remains largely unclear. This article discusses the clinical course of COVID-19 in pediatric patients along with laboratory and radiologic findings with emphasis on cardiovascular complications and on pediatric patients with CHD.

## Congenital Heart Disease

**Figure 1:**
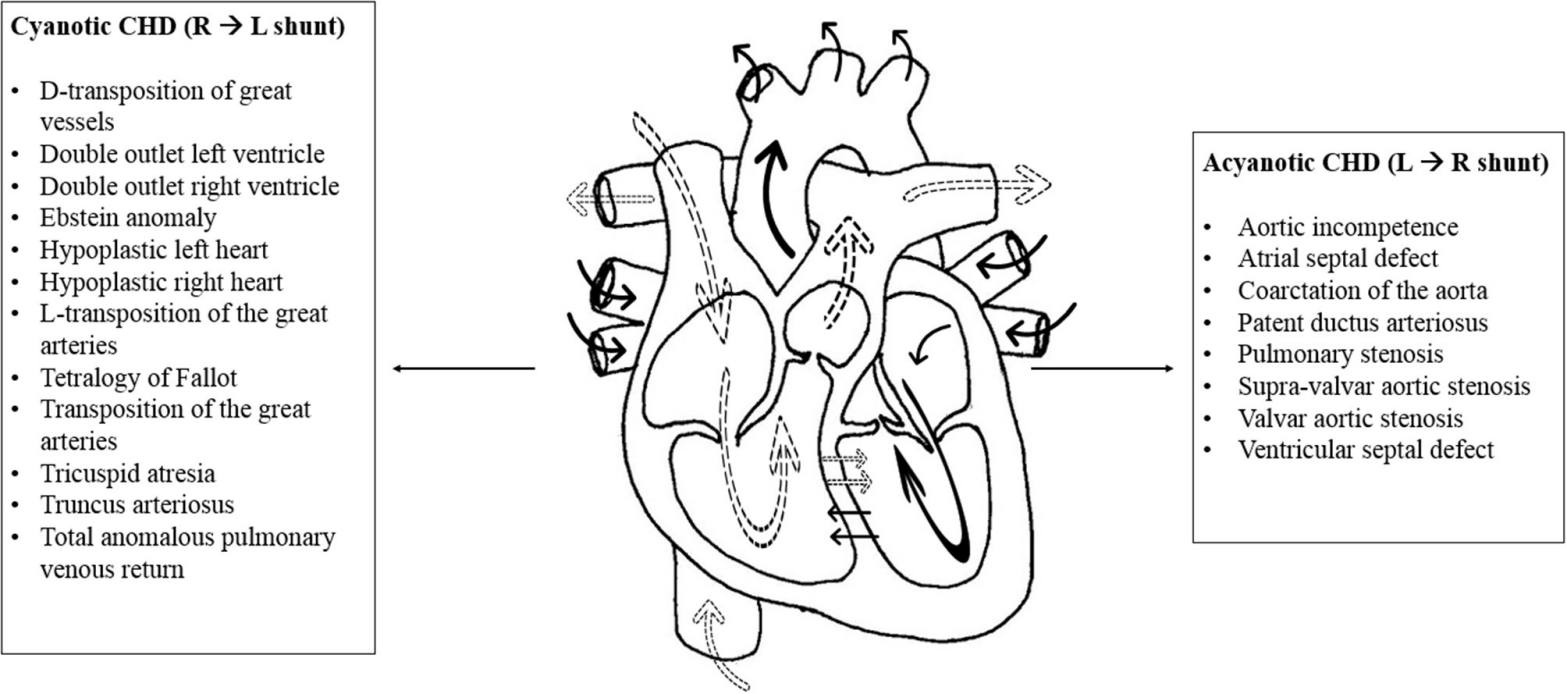
Cyanotic and acyanotic congenital heart diseases. The normal physiology of the heart is represented in the above illustration with the effect of shunts. The dotted arrows represent deoxygenated blood while solid arrows represent oxygenated blood. Shunt is an abnormal communication between the right and left sides of the heart or between the pulmonary and systemic circulations. The cyanotic congenital heart diseases are listed to the left of the diagram. In general, cyanotic lesions cause shunting of deoxygenated blood from the right to the left side of the heart, leading to decreased systemic oxygen saturation and cyanosis. Hence, they present early in life. Acyanotic congenital heart diseases are listed to the right of the diagram. Acyanotic lesions cause left to right shunting of oxygenated blood. They increase blood flow to the pulmonary circulation without affecting oxygen saturation in the systemic blood (11, 12).

CHD is the most commonly encountered congenital anomaly accounting for around 30% of all congenital defects (8). Its incidence varies greatly among populations with average of 8 per 1,000 annual live births (9). In recent decades, the incidence of CHD increased due to development in screening and detection methods (8, 10). CHD is classified into two main categories: cyanotic and acyanotic heart diseases [Figure 1; (11)]. The acyanotic heart diseases represent the milder types of CHD. They include ventricular septal defects, atrial septal defects, pulmonary stenosis, aortic stenosis among others. On the other hand, the group of cyanotic CHDs usually presents with severe illness early in life. They include Tetralogy of Fallot, hypoplastic right heart, hypoplastic left heart, total anomalous pulmonary venous return among others (11, 12).

With the rapid development in detection methods and surgical techniques, the survival rate of CHD patients significantly improved. It greatly hinges on the type and severity of CHD, where in the milder diseases survival to adulthood reached almost 98% (13). However, these children remain at remarkable risk for increased morbidity and mortality from lower respiratory tract infections (14). For instance, respiratory syncytial virus infection is associated with increased risk of hospitalization, intensive care unit (ICU) admission, and mechanical ventilation requirement in these patients (3). Consequently, it is critical to assess the burden of COVID-19 pandemic on CHD patients. Unfortunately, this remains challenging ought to the low number of reported cases. This article summarizes the available evidence that describes the characteristics of COVID-19 in CHD patients.

## SARS-CoV-2 IN Pediatric Patients

In the early phase of the global pandemic, the reported number of infected children was low. As the disease progressed, more cases were reported. Later on, It has been noted that children are less susceptible to COVID-19 and if infected the majority display mild to no symptoms (15). Eventually, attention has drifted toward the pediatric population to explore the disease features and discover their role in spreading the virus. In fact, out of the first 11,791 cases diagnosed in China, only 74 cases were children below 18 years old (16). The first reported pediatric case was on January 20, 2020 (16, 17). Besides, it is estimated that pediatric cases account for around 1–5% of the total reported cases worldwide (18). In the below section we review the clinical features, laboratory and radiologic findings, and treatment of COVID-19 in the pediatric population.

### Clinical Features

Reported disease severity of infected children range from asymptomatic to mild, moderate, severe and critically ill cases (Table 1) depicts the clinical features of cases classified with different disease severity. Interestingly, gastrointestinal manifestations could be the initial and even the only symptoms of SARS-CoV-2 infection in children (24). Indeed, China's first critically ill pediatric case displayed only gastrointestinal symptoms in early disease state and then progressed rapidly to acute respiratory distress syndrome, septic shock, and renal failure (24). Most of the infected pediatric patients are asymptomatic, or exhibit mild disease, usually recovering in 2 weeks from the onset of symptoms (17, 19, 23). In concordance, out of 171 cases in a Chinese study around 15% were clinically asymptomatic with negative CT chest findings (25).

**Table 1:** This table summarizes the clinical features encountered in pediatric COVID-19 patients according to disease severity and as described by various studies (15, 19–23).

**Severity**	**Clinical features**
Asymptomatic	Positive PCR test without clinical manifestations or radiologic findings.
Mild	Low- or high-grade fever, fatigue, myalgia, sore throat, headache, sneezing, dry cough, runny nose, nasal congestion, and gastrointestinal symptoms such as nausea, vomiting, abdominal pain, diarrhea, and non-severe pneumoniae on chest imaging.
Moderate	Lower respiratory tract infection symptoms, fever, dry or productive cough, wheezing, and positive findings on chest imaging.
Severe	Rapid progression of the disease with dyspnea, central cyanosis, increased respiratory rate above 70/min for infants and above 50/min for children greater above the age of 1 year, coma, convulsions, somnolence, severe dehydration, and oxygen saturation below 92%.
Critically ill	Rapidly progressing to acute respiratory symptoms, respiratory failure and end organ damage such as heart failure, shock, acute kidney injury, liver injury, encephalopathy, coagulopathy, or myocardial damage.

Studies from different countries reporting pediatric cases highlight the mild to moderate disease presentation in this population. Pediatric cases from all ages were reported. In a report by Dong et al., out of 2,135 Chinese children diagnosed with COVID-19 more than 90% were classified in the asymptomatic, mild or moderate disease categories (15). In Turkey, more than 50% of infected children had mild disease (26). Moreover, compared to adults, pediatric cases are less likely to report the common COVID-19 symptoms (25, 27, 28). Among 291 confirmed pediatric cases from the United States, only 56, 54, and 13% reported fever, cough and shortness of breath, respectively (28). Similarly, out of the 171 confirmed pediatric cases in a Chinese study, only 41.5% reported fever (25).

While as mentioned above most cases are mild, severe and critically ill cases were also reported. Of the 171 cases in the Chinese study, 3 patients required pediatric intensive care unit (PICU) admission, and all had underlying medical illness (leukemia on chemotherapy, hydronephrosis and intussusception). Eventually one death was reported for the 10 months old patient with intussusception, at week 4 post-admission, due to multi-organ failure (25). Congruently, among 745 reported cases in the United States, 147 required hospitalization and of which 15 patients required ICU admission (28). Dong el al., reported that of the 2,143 COVID-positive children, around 5% had severe disease while 0.6% were critically ill (21).

Remarkably, the highest hospitalization rate among the pediatrics population was reported in patients aged <12 months, accounting for up to 62% of the total hospitalized COVID-19 positive children (28). Similarly, 32 and 10.6% of the severely ill children were <1 year old (21, 29). In a Turkish study, 80% of PICU admissions were <1 year old (26). The percentage of severely and critically ill patients decreased with age, it is estimated to be 7.3, 4.2, 4.1, and 3% in age groups 1–5 years, 6–10 years, 11–15 years, and 16–17 years, respectively (21).

Fortunately, no evidence of vertical transmission has been detected. All tested neonates (*n* = 10) born to COVID-19 mothers had negative PCR results, although adverse effects were reported (30). Furthermore, infected neonates tend to show mild to moderate clinical symptoms (31). Only 1 out of 3 neonates had complicated hospital course associated with disseminated intravascular coagulation (DIC) and required non-invasive ventilation, but gradually improved (31). However, this neonate was preterm born at 31 weeks of gestation with neonatal respiratory distress syndrome (31).

Children susceptible to develop severe illness are those with underlying cardiac, respiratory or immunologic diseases such as CHD, asthma or immunodeficiency. 77% (28/37) of hospitalized patients and 100% of the ICU admitted with known information about hospitalization and medical status had underlying medical condition (28).

In summary, children with COVID-19 usually exhibit mild disease with minority requiring hospitalization and PICU admission. Infants have the highest risk of developing severe and critically ill disease. In addition, children with underlying medical condition are more likely to experience complications requiring hospital admission. Remarkably, vertical transmission has not been documented. Infected neonates have mild illness unless complicated by underlying medical problems or by prematurity.

### Laboratory Findings

Strangely, the majority of infected children tend to have normal laboratory markers including CRP and liver function enzymes (32, 33). In one study, procalcitonin and CK-MB elevation was reported in 16/20 and 15/20 of infected children, respectively (32). Variability in lymphocyte counts was reported. The majority (around 70%) had normal lymphocyte count (34); however lymphocytopenia was reported in 2 studies accounting for 3 and 3.5% of the population (25, 34). Besides, increase in WBCs counts and leucopenia were both reported (23, 25, 35). Some laboratory markers found to be associated with severe disease including: decreased lymphocyte count and increased procalcitonin, d-dimer, and CK-MB (19, 23). Coinfection with other pathogens was documented. Such pathogens include influenza viruses A and B, respiratory syncytial virus (RSV), cytomegalovirus (CMV) among others (32).

### Radiologic Findings

Published data describing the radiographic findings in pediatric patients with COVID-19 is scarce. During routine practice, chest x-ray (CXR) is the preferred modality in children; however, it has low specificity and sensitivity in evaluating lung involvement in confirmed or suspected COVID-19 children (36). In general, children have lower incidence and limited lung involvement compared to adults, as evident on chest imaging (37, 38). Ground glass opacities (GGO) with peripheral distribution in the lower lung fields is the most common reported finding (37, 39–41). Besides, the extent of pulmonary changes on imaging is related to disease severity. Patients with mild disease presentation often exhibit no radiographic changes; however, in one study, GGO were detected in 100% of patients with moderate COVID-19 (23).

More serious changes were seen in patients admitted to the PICU (42). In a study describing eight PICU patients, seven had multiple patch-like shadows, six had GGO, and one had pleural effusion and white lung-like change (42). These changes persisted even after resolution of clinical symptoms. Lesions may also persist in the absence of viral detection on PCR testing (32).

Mixture of findings with heterogenous pattern were reported on chest CT. Among the reported changes are bilateral or unilateral ground glass opacities, patchy ground glass opacities, local and bilateral patchy shadowing, interstitial abnormalities, halo signs, small nodular ground glass opacities, and speckled ground glass opacities, and bronchial pneumonia-like changes (25, 39–41, 43, 44). Rarely, pleural effusion, crazy-paving sign, and lymphadenopathy were reported but were witnessed mainly in severe cases (37, 39, 41). Due to the high radiation associated with CT scan and to the fact that most children experience milder lung involvement than adults, it is suggested that chest CT should not be routinely used unless necessary (45).

### Treatment

Most studies and trials targeting COVID-19 treatment were performed on adults. In general, in the pediatric population, treatment is symptomatic and supportive. It aims to provide rest, sufficient caloric intake, and maintaining water and electrolyte balance (19). It includes antipyretics for fever, sedatives in case of seizure, oxygen therapy including mask, nasal catheter, high flow nasal cannula or invasive ventilation, and antibiotics for bacterial superinfection (19, 46). In some cases, antiviral therapies are used as well (47).

Ultimately, vaccination remains the optimal preventive measure that can attenuate the global propagation of COVID-19. Researchers worldwide are working with unpreceded efforts to develop an effective vaccine. Currently, 42 vaccine candidates are being tested in pre-clinical or clinical trials (phases 1–3). Nevertheless, on the 11th of August 2020, the first COVID-19 vaccine has been granted approval. This vaccine stands for the Sputnik V vaccine developed by the Gamaleya Research Institute in Moscow. However, the use of this vaccine has raised a lot of arguments since it has not been tested in phase 3 clinical trials yet (48, 49). Hence, further trials are definitely needed to evaluate the role of any vaccine targeted against SARS-CoV-2.

## Cardiac Manifestations of Covid-19 in Pediatric Patients

In previously healthy COVID-19 pediatric patients, Kawasaki-like disease and myocarditis have been the main cardiovascular manifestations of SARS-CoV-2. These manifestations are triggered primarily by the massive immune response mounted against the viral infection (50–55). In fact, elevated levels of inflammatory markers have been noted in patients with COVID-19 associated Kawasaki-like disease or myocarditis (52, 54, 56). Il-6 was found to be inappropriately elevated in two distinct cases of pediatric myocarditis (52, 54). Similarly, CRP, pro-calcitonin and ferritin were elevated in most cases of Kawasaki-like disease and myocarditis (54, 56–58). Kawasaki-like disease may occur following a prior infection of COVID-19 documented by the presence of SARS-CoV-2 IgG antibodies or a known contact with a confirmed case of COVID-19 (58). This suggests the presence of a post-infectious state of immune dysregulation in children previously infected with or exposed to COVID-19 (51, 57).

Besides, compromised lung function may result in compromised cardiac function; this is attributed to numerous COVID-19-induced pulmonary defects denoted by oxidative stress, tissue injury, respiratory failure and ventilation perfusion mismatch (50, 51). Cardiac dysfunction may be possibly caused by direct myocardial damage. SARS-CoV-2 can gain entry to the cardiomyocytes through the ACE2 receptor (59). Subsequently, myocardial infiltration by SARS-CoV-2 and inflammatory cells may result in lethal complications that include fulminant myocarditis and cardiogenic shock (59).

After several cases were reported, the Kawasaki-like disease induced by COVID-19 was defined by the Centers for Disease Control and Prevention (CDC) as the multisystem inflammatory syndrome in children (MIS-C) associated with COVID-19 (60). As of the 15th of July, 342 cases of MIS-C and 6 deaths were declared in 37 American states (60). Eighty-one percent of the cases were aged between 1 and 14 years. Nevertheless, the disease may develop 2–4 weeks after a COVID-19 infection in any patient aged <21 years (60). SARS-CoV-2 infections complicated by MIS-C are often associated with fatal conditions denoted by heart failure, hepatic injury, renal dysfunction and coagulopathies (56).

Furthermore, in a cohort of 58 pediatric patients with MIS-C, 50% of the patients were admitted to the PICU (58). Acute kidney injury was noted in 13 patients, and cardiogenic shock necessitating inotropic agents was experienced by 27 patients (58). Of these 58 patients, 25 were bound to mechanical ventilators (58). Similarly, in a cross-sectional study of 48 patients with severe COVID-19 disease requiring PICU admission, death was witnessed only in two patients (61). Multi-organ dysfunction was the cause of death in both of them. Congruently, this endorses in turn the presence of a state of hyperinflammation in critically ill patients with multi-organ involvement (61). Cases of MIS-C have been reported in further studies as depicted in Table 2. To date, Kawasaki-like manifestations and myocarditis have constituted the key clinical presentations of this syndrome as revealed by most studies.

**Table 2:** This table summarizes the clinical characteristics of children with multisystem inflammatory syndrome reported in a few additional clinical studies.

**Study**	**Date of publication**	**Study type**	**Country**	**Number of patients**	**Age/Age range**	**Highlights/Findings**
Cardiac Dysfunction and Shock in Pediatric Patients With COVID-19 (62)	15 July 2020	Case Series	USA	3	6–13 years	• A picture of multisystem inflammatory syndrome was seen in the three patients • Ventricular dysfunction and cardiac shock were among the encountered complications • The three patients were admitted to PICU and discharged few days after admission
Kawasaki-like multisystem inflammatory syndrome in children during the covid-19 pandemic in Paris, France: prospective observational study (63)	3 June 2020	Prospective observational study	France	21	3.7–16.6 years	• A picture of multisystem inflammatory syndrome was seen in all patients • Myocarditis was noted in 76% of the patients • SARS-CoV-2 infection was detected in 19 patients • Gastrointestinal symptoms were reported by all patients • IVIG was given to all patients, and corticosteroids to 10 of them
COVID-19–Associated Pediatric Multisystem Inflammatory Syndrome (64)	22 May 2020	Letter to editor	USA	1	6 years	• A 6-year-old female patient presented with symptoms of fever, sore throat and decreased oral intake. She was initially diagnosed as group A streptococcus pharyngitis • Her condition was complicated by respiratory distress, cardiac failure, and electrolytic abnormalities (i.e., Hyponatremia, hyperkalemia, and azotemia) • In short, she had findings suggestive of Kawasaki-like disease and myocarditis, and was admitted to PICU • After a week of illness, COVID-19 PCR was found to be positive
Multisystem Inflammatory Syndrome in Children in New York State (65)	23 July 2020	Retrospective observational study	USA	191	0–20 years	• Of the 191 patients, 95 had confirmed MIS-C • Fever was noted in all patients • CRP was elevated in all patients. D-dimer and troponin were found elevated in 91 and 71% of the patients • Vasopressors were given to 62% of the patients • 80% of the patients were transferred to ICU • 2 deaths were reported
Multisystem Inflammatory Syndrome in U.S. Children and Adolescents (66)	23 July 2020	Observational study	USA	186	0–20 years	• A picture of multisystem inflammatory syndrome was seen in all patients • Gastrointestinal symptoms followed by cardiovascular symptoms were reported in most patients • 80% of the patients were admitted to ICUMechanical ventilation and vasopressors were provided to 20 and 48% of the patients, respectively• Inflammatory markers were elevated in almost all patients • 4 deaths were reported
Outbreak of Kawasaki disease in children during COVID-19 pandemic: a prospective observational study in Paris, France (67)	14 May 2020	Prospective observational study	France	17	3.7–16.6 years	• A picture of Kawasaki disease was seen in all patients • Gastrointestinal symptoms were initially reported by all patients • Inflammatory markers were elevated in all patients • 11 patients were admitted to ICU • 12 patients had myocarditis • No deaths were reported
Multisystem Inflammatory Syndrome in Children (MIS-C) Related to COVID-19: A New York City Experience (68)	25 June 2020	Retrospective observational study	USA	15	3–20 years	• A picture of MIS-C was seen in 15% of patients • Not all patients had positive PCR tests, but all had positive serology suggesting post-infection inflammatory response rather than direct viral injury • Initially, lymphopenia (in 13 patients), thrombocytopenia (6), hypoalbuminemia (8), and elevated fibrinogen (14) were noted • Inflammatory markers were elevated: CRP and D-dimer (100% of patients), ferritin (87%), and ESR (93%) • Interleukin-6 and interleukin-8 were elevated in all patients • 13 patients had gastrointestinal symptoms while only 5 patients reported respiratory symptoms • 13 patients showed features of severe cardiac involvement • 9 patients required vasopressors or inotropes, and 1 patient required intra-aortic balloon pump • 20% required intubation, 33% non-invasive ventilation • 1 death in a child who required extra-corporeal membrane oxygenation for 9 days
Acute heart failure in multisystem inflammatory syndrome in children (MIS-C) in the context of global SARS-CoV-2 pandemic (69)	17 May 2020	Retrospective observational study	France and Switzerland	35	2–16 years	• 31/35 had positive PCR tests • 28% had comorbidities • Gastrointestinal symptoms were reported in 80% • 33% had left ventricular ejection fraction below 30%, 72% below 50%, 80% had cardiogenic shock, 3% ventricular arrhythmia, 17% coronary artery dilation • 100% required ICU admission • 80% needed inotropic agents and 28% needed ECMO • Elevated BNP, troponin, CRP, and D-dimer was documented • IL-6 elevated in 13 patients • Left ventricular function returned to normal in 25 patients in 7 days • No deaths were reported
Systemic Inflammation With Cardiac Involvement in Pediatric Patients With Evidence of COVID-19 in a Community Hospital in the Bronx, New York (70)	20 July 2020	Case Series	USA	4	3–20 years	• All patients had initial negative PCR tests but positive serology • 3 patients had gastrointestinal symptoms, all had fever, and none had respiratory symptoms • Elevated CRP, ferritin, troponin and pro-BNP noted in all patients, D-dimer in 3 patients and fibrinogen in 2 patients • 3 patients had mild and 1 severe depression of left ventricular function • 1 patient required intubation • 1 patient developed vasogenic shock required ECMO and died due intracranial hemorrhage associated with herniation
COVID-19 and Kawasaki Disease: Novel Virus and Novel Case (71)	7 April 2020	Case report	USA	1	6 months	• A 6-month-old baby girl presented with a picture of Kawasaki disease • COVID-19 PCR was found to be positive • Inflammatory markers (i.e., CRP and ESR) were elevated • No cardiac abnormalities were noted • Patient was treated with IVIG and aspirin
SARS-CoV-2-related pediatric inflammatory multisystem syndrome, an epidemiological study, France, 1 March to 17 May 2020 (72)	4 June 2020	Case series	France	156	5–11 years	• Cases were classified into: confirmed/proven SARS-Cov-2 related pediatric inflammatory multisystem syndrome (CoV-PIMS) (79 patients), probable CoV-PIMS (16), possible CoV-PIMS (13), and non—CoV-PIMS (48) • Myocarditis and Kawasaki-like disease were noted in 70 and 61% of the CoV-PIMS, respectively • Macrophage activation syndrome and seritis documented in 25 and 24% of the CoV-PIMS, respectively • 67% of the CoV-PIMS required ICU admission, of these 43% required intubation and mechanical ventilation, and 73% needed vasopressors or inotropes • 1 death was reported

Finally, despite being less common, arrhythmias were also encountered in children with COVID-19. As compared to adult patients, milder forms of arrhythmias were reported in pediatric patients. In one pediatric study, ventricular tachycardia and ventricular fibrillation were not reported (32). Yet, sinus tachycardia, atrial tachycardias, atrial and ventricular premature beats, bundle branch blocks, and first-degree AV block were the main arrhythmic manifestations of COVID-19 among the studied patients (32).

## COVID-19 IN Pediatric CHD Patients

**Figure 2:**
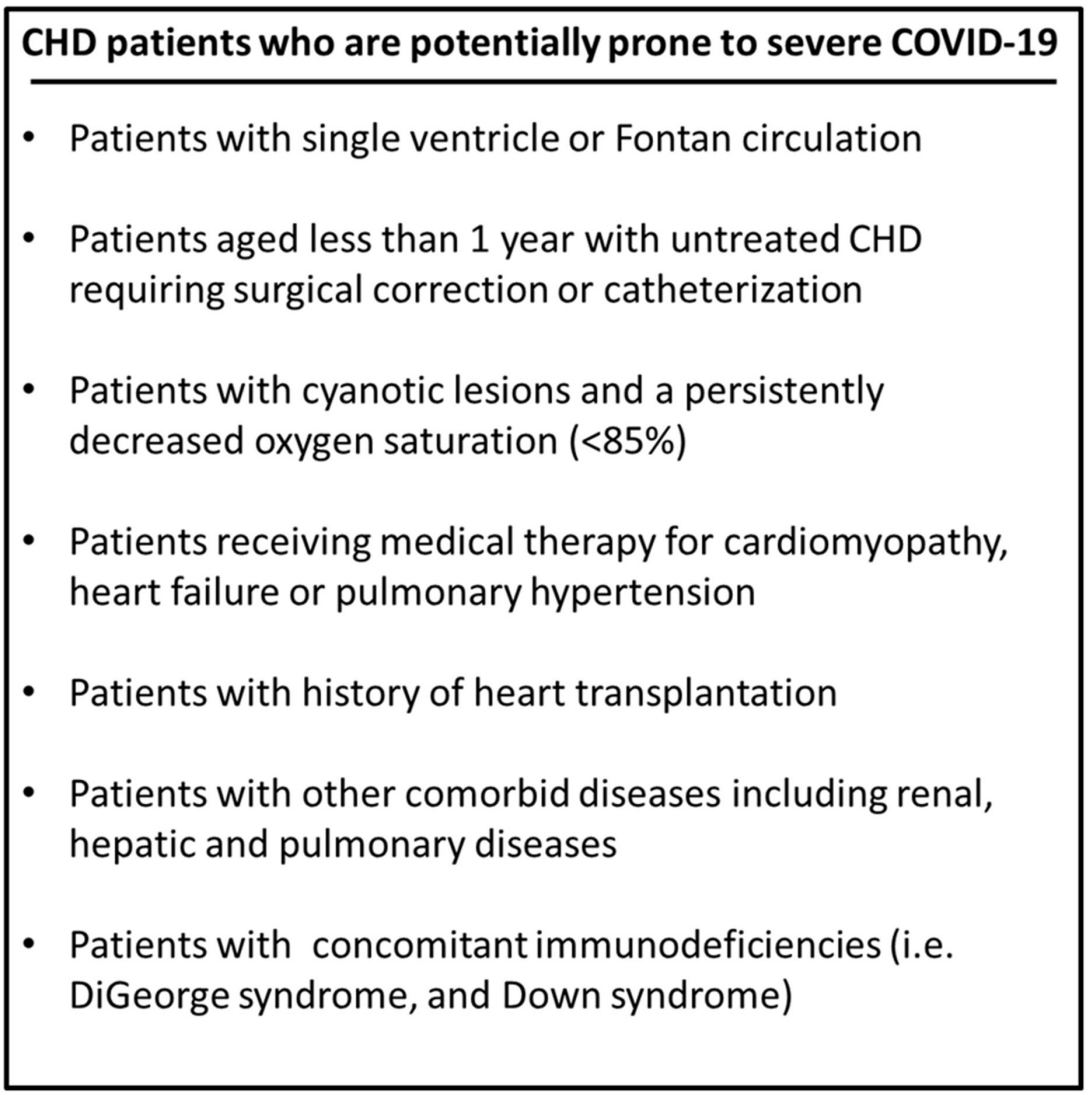
Patients with complex CHD who are considered at high-risk of developing severe COVID-19 as per the British Congenital Cardiac Association (BCCA).

As previously mentioned, children are less prone to acquiring COVID-19. Additionally, as compared to adults, they often exhibit mild disease ranging from flu-like to no symptoms. Nonetheless, children, particularly those with CHD, may develop serious COVID-19 related cardiovascular complications (51, 73). CHD patients are more likely to require ICU admission and artificial respiratory support, especially those with cyanotic defects. In these patients, COVID-19 may lead to worsened hypoxemia and compromised tissue perfusion (51, 73). Additionally, patients with complex CHD complicated by depressed myocardial contractility, pulmonary hypertension, immunodeficiencies (i.e., DiGeorge syndrome) among other comorbid conditions may likely develop severe and critical COVID-19 disease (73, 74). Indeed, as per the British Congenital Cardiac Association (BCCA), CHD patients and particularly those with complex disease (Figure 2) are considered at-risk patients prone to develop severe COVID-19 (75).

Despite the scarcity of available evidence in this area, few studies have reported the complications encountered in SARS-COV-2 positive pediatric cardiac patients. In this context, *Lu et al*. declared that severe disease was mainly witnessed in children with preexisting life-threatening conditions. Out of 230 patients, two had severe symptoms. One child had a past medical history of surgically treated CHD. The other had complicated kidney disease (22).

Furthermore, in a former Chinese study published on February 2020, Chinese experts have discussed the characteristics of the COVID-19 pandemic along with its diagnosis, treatment and prevention in children aged up to 17 years. They defined CHD as a risk factor for critical SARS-CoV-2 infection (76). This has been likely endorsed by a retrospective study of 25 Chinese children aged between 3 months and 14 years. In this study, serious and complicated COVID-19 was observed only in two patients having a past medical history of surgically treated CHD (77).

Additionally, in a cross-sectional study of 48 participants, Shekerdemian et al. assessed the clinical presentation, characteristics, and outcomes of COVID-19 pediatric patients admitted to ICU in North America. In this study, underlying comorbid conditions were noted in 83% of the patients. A History of CHD was observed in 3 patients (61). Similarly, in a multi-center observation study, data concerning COVID-19 in CHD patients was collected from eight Italian CHD centers (74). Throughout a period of 6 weeks extending from the 21st of February till the 4th of April, a total of 76 SARS-CoV-2 positive CHD patients were reported. Four were children and the remaining 72 were adults aged more than 18 years. The reported cases were likely subdivided into confirmed (9 patients) and suspected COVID-19 cases (67 patients) (74). Cardiovascular complications, such as heart failure, arrhythmias, stroke, myocardial injury, pericardial effusion and pulmonary hypertension were mainly observed in the confirmed cases. Nonetheless, a mild disease course was witnessed in most patients and zero deaths were reported (74).

As deduced from above and just like in healthy individuals, COVID-19 may exhibit distinct clinical courses in CHD patients ranging from no symptoms to critical disease. Nonetheless, COVID-19 manifestations such as chest pain, cyanosis, dyspnea, and palpitation may imitate symptoms of cardiovascular deterioration in these patients (73). Hence, comprehensive evaluation and meticulous care should be offered to any CHD patient presenting with these symptoms that may indicate worsening CHD or new onset SARS-CoV-2 infection. Additionally, the diagnosis of COVID-19 in CHD patients is made primarily through RT-PCR testing of nasopharyngeal samples (51, 59). The detection of suggestive chest CT scan findings plays likely an imperative role in confirming the diagnosis of COVID-19 in suspected cases (51, 59).

In most cases, patients with CHD are managed with supportive measures geared toward fever reduction, symptoms control and oxygen correction. The use of repurposed medicines such as azithromycin, hydroxychloroquine, dexamethasone, remdesivir, and immunotherapies is not part of the standard management (51, 78). Yet, the addition of these medications to the management of CHD patients necessitates careful dosing and thorough monitoring particularly when dealing with cardiotoxic medications (51, 78). Besides, none of the medications that are often used in treating CHD manifestations and complications, and also in maintaining cardiac functions in these patients are shown to affect or exacerbate the clinical course of COVID-19 in this population (73). For instance, the discontinuation of angiotensin converting enzyme inhibitors (ACE-i) and angiotensin II receptor blockers (ARB) in cardiac patients with confirmed or suspected COVID-19 was prohibited by the Heart failure Society of America (HFSA), American College of Cardiology (ACC), American Heart Association (AHA), and the European Society of Cardiology (ESC) (79, 80).

Finally, numerous studies have emphasized the importance of COVID-19 prevention in this population (51, 59, 73, 78, 81). Indeed, effective screening and early detection of SARS-CoV-2 infection in patients with CHD are key for avoiding severe life-threatening manifestations of the disease. Congruently, CHD patients should be educated about the signs and symptoms of COVID-19, and also about the importance of adopting protective measures such as social distancing, repeated hand washing, and effective wearing of face masks and goggles (51, 59, 73, 78, 81).

## Limitations

The studies reported in our manuscript have several limitations. First, they are restricted in terms of population size. Most reported studies were from single-centers and had a small number of pateints. Second, no randomized controlled trials were found; the studies were mostly observational, case series and retrospective chart reviews. Hence, conclusive evidence concerning infection rate among various pediatric age groups, genders and those with comorbidities cannot be ascertained.

Similarly, the literaure lacks studies comparing COVID-19 infection in heathy children to those with CHD. In addition, the published data regarding children with multisystem inflammatory syndrome is limited by the fact that patients were only from 3 countries. Seven out of the eleven included studies were from centers across the United States, three were from France and one had patients from both France and Switzerland. Besides, the studies were heterogenous in terms of the assessed laboratory markers. Finally, data describing laboratory values and imagings are scarce and highly variable among infected children with CHD. Despite these limitations, this manuscript was able to describe the spectrum of disease presentation and prognosis in CHD patients with superimposed COVID-19 infection.

## Conclusion

Little is known about the SARS-CoV-2 infection in CHD patients. The majority of the published data consist of documentaries, narrative reviews, and letters. Yet, as depicted above, it is now considered that serious clinical symptoms and end-organ complications of COVID-19 may develop in children with CHD or previous history of surgically treated CHD. Ultimately, as the COVID-19 pandemic continues to spread globally at enormous rates, further higher-quality clinical trials with enrolled pediatric CHD patients are required to assess the clinical burdens of COVID-19 in these patients. Similarly, internists, pediatricians, and cardiologists should understand the influence of this pandemic on CHD patients and should provide meticulous care to this at-risk population.

## Author Contributions

MA, FB, and AE developed the idea and the review framework. RZ and NY wrote the first draft of the manuscript. AE did the final editing. All authors contributed to corrections and adjustment of subsequent iterations of the manuscript. All authors approve and agree with the content.

## Conflict of Interest

The authors declare that the research was conducted in the absence of any commercial or financial relationships that could be construed as a potential conflict of interest.
